# Adjustable extracellular matrix rigidity tumor model for studying stiffness dependent pancreatic ductal adenocarcinomas progression and tumor immunosuppression

**DOI:** 10.1002/btm2.10518

**Published:** 2023-04-05

**Authors:** Haoxiang Zhang, Jiaoshun Chen, Xiaoqing Hu, Jianwei Bai, Tao Yin

**Affiliations:** ^1^ Department of Pancreatic Surgery, Union Hospital, Tongji Medical College Huazhong University of Science and Technology Wuhan 430022 China; ^2^ Sino‐German Laboratory of Personalized Medicine for Pancreatic Cancer, Union Hospital, Tongji Medical College Huazhong University of Science and Technology Wuhan 430022 China; ^3^ Department of Ultrasound Medicine, Union Hospital, Tongji Medical College Huazhong University of Science and Technology Wuhan 430022 China

**Keywords:** extracellular matrix rigidity, gelatin methacryloyl, hydrogels, pancreatic cancer, tumor immunosuppression

## Abstract

Pancreatic ductal adenocarcinomas (PDAC) is one of the stiffest malignancies with strong solid stresses. Increasing stiffness could alter cellular behavior and trigger internal signaling pathways and is strongly associated with a poor prognosis in PDAC. So far, there has been no report on of an experimental model that can rapidly construct and stably maintain a stiffness gradient dimension in both vitro and in vivo. In this study, a gelatin methacryloyl (GelMA)‐based hydrogel was designed for in vitro and in vivo PDAC experiments. The GelMA‐based hydrogel has porous, adjustable mechanical properties and excellent in vitro and in vivo biocompatibility. The GelMA‐based in vitro 3D culture method can effectively form a gradient and stable extracellular matrix stiffness, affecting cell morphology, cytoskeleton remodeling, and malignant biological behaviors such as proliferation and metastasis. This model is suitable for in vivo studies with long‐term maintenance of matrix stiffness and no significant toxicity. High matrix stiffness can significantly promote PDAC progression and tumor immunosuppression. This novel adaptive extracellular matrix rigidity tumor model is an excellent candidate for further development as an in vitro and in vivo biomechanical study model of PDAC or other tumors with strong solid stresses.

**Abbreviations**
3D: three dimensions
AJCC 8th: American Joint Committee on Cancer staging system
B: B cells
CAF: cancer‐associated fibroblasts
CCTCC: China Center For Type Culture Collection
CD4^+^ Th: CD4^+^ helper T cells
CVF: collagen volume fraction
DC: dendritic cells
DEG: differentially expressed gene
DoF: degree of functionalization
DS: degree of substitution
ECM: extracellular matrix
effect CD8^+^ Tc: effect CD8^+^ cytotoxic T cells
EHS: Engelbreth–Holm–Swarm
exhaust CD8^+^ Tc: exhaust CD8^+^ cytotoxic T
GelMA: gelatin methacryloyl
GSVA: gene set variation analysis
IF: immunofluorescence
IHC: immunohistochemistry
LAP: lithium phenyl‐2 4 6‐trimethylbenzoylphosphinate
M1: M1 macrophages
M2: M2 macrophages
Masson: Masson's trichrome
Naive T: endothelial Naive T cells
NK: natural killer cell
OS: over survival
PDAC: pancreatic ductal adenocarcinomas
SEM: scanning electron microscope
SPF: specific pathogen‐free conditions
TMA: tissue microarray
TME: tumor microenvironment
Treg: regulatory T cells

## 1 INTRODUCTION

Recently, the incidence of pancreatic cancer has shown a significant upward trend worldwide.^1^ Statistics in 2022 show that among all malignant tumors in the United States, pancreatic cancer ranks 10th in men and 8th in women and ranks 4th in cancer‐related mortality.^2^ The progression of pancreatic ductal adenocarcinomas (PDAC) is marked by significant fibrosis. It is well known that fibrosis can promote both pancreatic cancer proliferation, promote its metastasis, and block the uptake of chemotherapy drugs.^3^, ^4^

Extracellular matrix (ECM) is a three‐dimensional noncellular network structure that plays a vital role in maintaining the function and structure of an organization. Cell microenvironment disorders are caused by ECM synthesis, deposition, remodeling, cross‐linking, and enzyme modification. Deposition, remodeling, and cross‐linking of ECM composition can cause fibrosis to stiffen the stroma, thus exerting mechanical forces on the cells, known as stiffness or rigidity.^5^ PDAC has a large stromal component, making it one of the most stiff malignancies with solid stresses that exceed 10 kPa.^3^

While traditional views focus primarily on chemical properties, recent studies have demonstrated that physical properties contribute significantly to physiological and pathological outcomes of the ECM. The ECM has been studied for its stiffness, related to the slow elastic force on the cells. There was an increase in tumor stiffness in PDAC due to massive ECM deposition, particularly collagen and hyaluronic acid. Increasing stiffness could alter cellular behavior and trigger internal signaling pathways.^6^ Fibronectin is produced when the ECM becomes stiffer, binds to extracellular collagen, fibrin, heparan sulfate proteoglycans, and integrins. Increased stiffness of the ECM increases cell adhesion to the ECM, connects the ECM to the cytoskeleton through local adhesion proteins, and increases cytoskeletal tension through Rho/ROCK signaling.^7^ Integrin aggregation promotes tumor progression by recruiting focal adhesion signaling molecules, including FAK, Src, paxillin, Rac, Rho, and Ras.^8^

Additionally, stiffening the ECM increases PI3K activity and tumor invasion.^9^ The increase in matrix stiffness in PDAC promotes the activation of YAP/TAZ, which, in turn, stimulates the production of the ECM protein and profibrotic mediators.^10^, ^11^, ^12^ Additionally, ECM stiffness and oncogene‐mediated changes in cell mechanical properties are essential for reprogramming normal cells into tumor precursors.^13^

Gelatin methacryloyl (GelMA) has been widely used in various biomedical applications, because they possess suitable biological properties and a wide range of physical properties that can be tailored. As the hydrolysis product of collagen, the main component of ECM in most tissues, the properties of GelMA hydrogels in three dimensions (3D) closely resemble those of native ECM due to the presence of cell‐attaching proteins and matrix metalloproteinases.^14^, ^15^, ^16^ GelMA is also versatile from a processing perspective. In response to light irradiation, it forms a hydrogel with tunable mechanical properties that mimic the native ECM.

Matrix stiffness is strongly associated with poor prognosis and PDAC.^6^, ^17^ Increasing stiffness in pancreatic cancer tissue occurs slowly and gradually. So far, there has been no report on developing an experimental model that can rapidly construct and stably maintain a stiffness gradient dimension both in vitro and in vivo. In this study, by adjusting GelMA concentration and degree of substitution (DS), a novel adjustable extracellular matrix rigidity tumor model based on visible light (405 nm) cross‐linked hydrogels was designed for in vitro and in vivo PDAC experiments. The GelMA‐based hydrogel has porous, adjustable mechanical properties and excellent in vitro and in vivo biocompatibility. In vitro 3D culture method designed based on GelMA can effectively form a gradient and stable extracellular matrix stiffness, affecting cell morphology, cytoskeleton remodeling, and malignant biological behaviors such as proliferation and metastasis. Furthermore, in vivo evaluation confirmed that this model is suitable for in vivo studies with no significant toxicity. High matrix stiffness can significantly promote PDAC progression.

## 2 MATERIALS AND METHODS

### 2.1 Tissue microarray

The tissue microarray (TMA; HPanA180Su03, Shanghai Outdo Biotech Company, Shanghai, China) contained 90 pancreatic cancer tissues and paired para‐cancer tissues with information of clinical–pathological parameters. The baseline characteristics of patients in TMA were shown in Table S1. Masson staining was performed on human pancreas cancer TMA using the trichrome stain kit (Masson) (Sigma‐Aldrich). Quantification of collagen density on Masson staining was performed with Fiji ImageJ software (https://imagej.net/software/fiji/). According to the different quantification of collagen density of each sample, the samples were divided into three grades, low, mid and high, 30 samples each.

### 2.2 Cell lines and cell culture

The human PDAC cell lines PAN1 (with Kras G12D mutation) and CFPAC‐1 (with Kras G12V mutation), and BXPC3 (without Kras mutation), were obtained from the China Center for Type Culture Collection (CCTCC). Murine PDAC cell line, LSL‐Kras(+/G12D); LSL‐Trp53(+/R172H); Pdx1‐Cre (KPC; representing Kras G12D mutation and Trp53 R172H mutation) were a kind gift from Fudan University Shanghai Cancer Center. After obtaining the KPC cell line, we identified the type of gene mutation used for next‐generation sequencing (NGS) performed by Sangon (Sangon Biotech, Shanghai, China). All cell lines were cultured in Dulbecco Modified Eagle Medium (DMEM; BasalMedia, Shanghai, China) containing 10% fetal bovine serum (FBS; Biological Industries, Israel) and 1% penicillin/streptomycin (BasalMedia, Shanghai, China). Cells were incubated in a CO_2_ incubator at 37°C in 5% CO_2_. Cells were tested to confirm the absence of Mycoplasma contamination using the MycoBlue Mycoplasma Detector (Vazyme, Nanjing, China).

### 2.3 GelMA hydrogels preparation

To generate hydrogels, 60% DS GelMA (GM60; EFL‐GM‐60, Yongqinquan Intelligent Equipment Co., Ltd., Suzhou, China) was resuspended at 5% and 20% (w/v) in PBS and incubated in a 55°C water bath until it was completely dissolved. 90% DS GelMA (GM90; EFL‐GM‐90, Yongqinquan Intelligent Equipment Co., Ltd., Suzhou, China) was resuspended at 10% (w/v) in PBS and incubated in a 55°C water bath until it was completely dissolved. The GelMA solution was then combined with the photoinitiator lithium phenyl‐2, 4, 6‐trimethyl‐benzoyl phosphinate (LAP) at a final concentration of 0.025% w/v. Finally, the solution was heated to 37°C in a water bath, followed by sterilization using a 0.22‐μm filter, and then aliquoted and stored at −20°C.

### 2.4 Rheological measurement

The rheological properties of 5% GM60, 10% GM90, and 20% GM60 hydrogel were explored on a rotating rheometer (TA‐DHR‐2TA, Instruments). To study the cross‐linked behavior of visible light (405 nm) cross‐linked behavior of the hydrogel, cross‐linked visible light (UV405 nm, 30 s, 25 mw/cm^2^) was performed in 30–60 s. The storage modulus (*G*′) was measured at a fixed angular frequency of 5 rad/s at 37°C with constant 1% strain.

### 2.5 Microstructure of the hydrogels

Five percent GM60 (1 Kpa), 10% GM90 (10 Kpa), and 20% GM60 (20 Kpa) were poured into Teflon molds and cross‐linked to hydrogels by a 405 nm light‐curing unit (EFL‐LS‐1601‐405, Yongqinquan Intelligent Equipment Co., Ltd., Suzhou, China) in 30 s. The sample was freeze‐dried. After spraying a thin gold film on the freeze‐dried hydrogel sample, the sample morphology was observed by SEM (TESCAN MIRA LMS, Czech Republic).

### 2.6 3D cell culture based on visible‐light (405 nm) cross‐linked hydrogels

Five percent GM60 (1 Kpa), 10% GM90 (10 Kpa), and 20% GM60 (20 Kpa) kept at 37°C in a metal bath, protected from light. We applied two methods for 3D cell culture in vitro (Protocol A and B).

#### 2.6.1 Protocol A

Cells were fully mixed with the GelMA solution and added dropwise to a 405 nm light cure unit (EFL‐LS‐1601‐405, Yongqinquan Intelligent Equipment Co., Ltd., Suzhou, China) covered with parafilm (antiadhesion). Cell climbing slices were then mounted on a drop of cell suspension. GelMA‐cell suspensions were cross‐linked into hydrogels by the light‐curing unit in 30 s. Gently removed the cell climbing slices and placed them into Petri dishes. A growth medium was added as usual (Figure 3a).

#### 2.6.2 Protocol B

Cells were fully mixed with the GelMA solution and added dropwise to Petri dishes. GelMA‐cell suspensions were cross‐linked into hydrogels by the light‐curing unit in 30 s and growth medium was added as usual (Figure 3b).

### 2.7 Biocompatibility and cytotoxicity test

Calcein/PI Cell Viability/Cytotoxicity Assay Kit (C2015M, Beyotime Biotechnology, Shanghai, China) was used to evaluate the biocompatibility and cytotoxicity of 3D cell culture with adjustable extracellular matrix rigidity (1, 10, and 20 Kpa) based on GelMA. Cells were collected and re‐seeded in 3D culture in a 24‐well plate following Protocol A as described above. After 5 days of cell culture, following the manufacturer instructions of manufacturer, cell climbing slices were co‐stained with Calcein/PI for 30 min at 37°C. Cell climbing slices were taken out and conformally sealed to the slide. Cell survival was observed using a confocal microscope (FV3000, Olympus, Japan), and 3D cell images were reconstructed. Dead/live cells were then counted by FIJI ImageJ software (https://imagej.net/software/fiji/).

### 2.8 Cell morphology, proliferation, and migration assay

The plate‐cloning assay was used for cell proliferation and migration assay. Cells were collected and reseeded in 3D culture in a 6‐well plate following Protocol B as described above. The morphological change of cells was observed under an inverted microscope. The formula calculated cell volume: volume = 3/4 × π(3.14) × length × width.^2^ After 5 days, the hydrogel was removed from the 6‐well plate. Cells that migrated from the hydrogel and adhered to a 6‐well plate for 5 days of culture were fixed and stained with crystal violet. The visible colonies were then counted using FIJI Image J software (https://imagej.net/software/fiji/).

### 2.9 Western blot analysis based on a 3D cell culture system

Protein extraction in a 3D cell culture system was called tissue protein extraction. Cells were collected and reseeded in 3D culture in a 6‐well plate following Protocol A as described above. After 5 days of cell culture, the media was removed and washed with PBS (2 × 2 mL). Use a cell spatula to gently lift the hydrogel and place it in a 1.5 mL microcentrifuge tube.

The samples were then lysed in RIPA lysis buffer and fully ground by hand using an electric grinding rod. After sonication and centrifugation at 12,000*g* for 15 min at 4°C, the supernatants were collected. The procedure remainder was performed as previously described.^18^

### 2.10 Immunofluorescence

Cells were collected and reseeded in 3D culture in a 24‐well plate following Protocol A as described above. After 5 days, cells in 3D cell culture were isolated by GelMA Lysis Buffer (EFL‐GM‐LS‐001, Yongqinquan Intelligent Equipment Co., Ltd., Suzhou, China) as described and seeded in climbing films. After adhering to the climbing films, cells were washed with PBS and fixed with 4% paraformaldehyde. The cytoskeletal organization was evaluated using Actin‐Tracker Green (C1033, Beyotime Biotechnology, Shanghai, China) and Tubulin‐Tracker Red (C1050, Beyotime Biotechnology, Shanghai, China) according to the manufacturer instructions. Cytoskeletal was observed by a confocal microscope (FV3000, Olympus, Japan).

Fluorescence intensities were analyzed using FIJI ImageJ software (https://imagej.net/software/fiji/).

### 2.11 In vivo experiments

All experiments involving animals in this research followed the ethical standards established by the Institutional Animal Care and Use Committee of Tongji Medical College, Huazhong University of Science and Technology. The formula calculated tumor volume: volume = 0.5 × length × width.^2^

### 2.12 Adjustable extracellular matrix rigidity orthotopic tumor transplantation murine models of PDAC in immune‐competent C57BL/6J mice

Eight‐week‐old male C57BL/6J were purchased from Beijing Weitong Lihua Experimental Animal Technology Co., Ltd. The animals were kept at the Experimental Animal Center of Tongji Medical College, Huazhong University of Science and Technology (Wuhan, China), under specific pathogen‐free conditions (SPF). Briefly, C57BL/6 mice (*n* = 8 in each group) were anesthetized with an intraperitoneal injection of 1.25% tribromoethanol (20 μL/g mice). Fur was shaved in the left‐flank surgical field using an electric clipper and cleaned up with a small vacuum cleaner. The incision region was sterilized with 75% alcohol. 5% GM60 (1 Kpa), 10% GM90 (10 Kpa), and 20% GM60 (20 Kpa) kept at 37°C in a metal bath, protected from light. KPC cells were mixed fully with GelMA solution (5 × 105 cells/20 μL) and injected into the pancreas body, which was exposed under the left flake incision. GelMA‐KPC cell suspensions were crosslinked into hydrogels by a 405 nm light‐curing unit (EFL‐LS‐1601‐405, Yongqinquan Intelligent Equipment Co., Ltd., Suzhou, China) in 30 s. The syringe needle was pulled out after being cross‐linked, and the abdominal wall was closed in layers with a 7–0 silk suture.

### 2.13 Imaging procedure

Elastography was performed using the SuperSonic Aixplorer ultrasound system (France) with an SL15‐4 linear transducer. When the pancreatic tumors are detected by echography, a study box is placed and a US wave is applied at different depths, compressing the tissue. Three elastographic images obtained in the maximal diameter plane were taken of each lesion in the elastography mode of SWE. SWE values were determined by setting fixed regions of interest (ROIs) on the entire lesion and adjacent tissue. All SWE values were recorded and the mean maximal SWE values were used for assessment.

### 2.14 Bulk RNA‐seq assay

The bulk RNA‐seq assay was performed by BGI (BGI, Beijing, China). Total RNA was extracted from murine tumor tissues by Trizol and sequenced at MGISEQ2000. The reference genome was Mus_musculus.GRCm38. After the performance and quality of the RNA‐seq was evaluated, a standard analysis pipeline was adopted to detect biological signals from the samples. The. Differentially expressed gene (DEG) analysis and functional enrichment were performed using the RNAseqStat2 package (Zeng J, Xia Y (2022). RNAseqStat2: A Pipeline to Process RNAseq Data. R package version 0.1.0.9993). Gene set variation analysis (GSVA) using the GSVA package (1.42.0) based on Hallmark gene sets (mh.all.v2022.1.Mm.symbols.gmt).

### 2.15 Space transcriptome

The space transcriptome (ST) data reported in this manuscript were obtained from published sources (GSE111672).^19^ Seurat clustering was used Seurat R package (version 4.3.0). The carcinoma foci in the HE staining pictures were observed and analyzed by two professional pathologists according to the lists of cell gene markers (Table S2). Signatures of PDAC tumor cells, epithelial cells, cancer‐associated fibroblasts (CAF), endothelial, Naive T cells (Naive T), CD4^+^ helper T cells (CD4^+^ Th), regulatory T cells (Treg), effect CD8^+^ cytotoxic T cells (effect CD8^+^ Tc), exhaust CD8^+^ cytotoxic T (exhaust CD8^+^ Tc), natural killer cell (NK), Dendritic cells (DC), monocyte cells, B cells (B), M1 macrophages (M1), M2 macrophages (M2) were constructed and scored through GSVA using the GSVA package (1.42.0).

### 2.16 Immunohistochemistry and immunofluorescence

Tumor tissues immunohistochemistry (IHC) and immunofluorescence (IF) were performed as previously described.^18^ The average integrated optical density (IOD) and fluorescence intensities in five randomly selected areas for each group were calculated using FIJI ImageJ software (https://imagej.net/software/fiji/).

### 2.17 Statistical analysis

Data were presented as mean ± SD. Statistical analysis was performed using GraphPad Prism 9.0 software via Student *t*‐test and one‐way ANOVA analysis. Differences were considered statistically significant at *p* < 0.05.

## 3 RESULT AND DISCUSSION

### 3.1 Matrix stiffness link to PDAC prognosis

**FIGURE 1:**
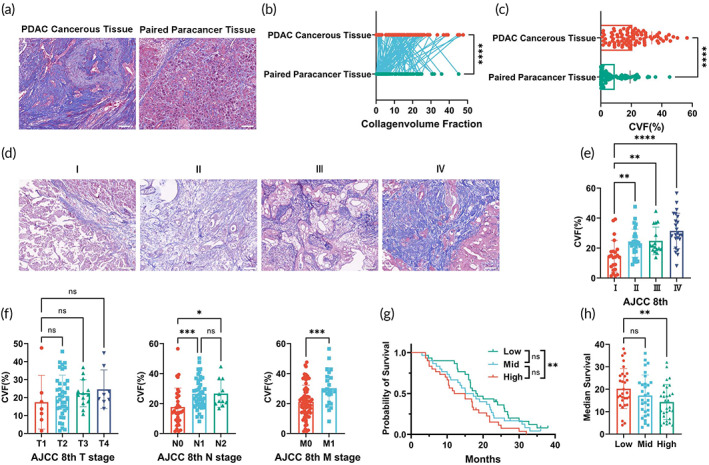
Matrix stiffness is related to the prognosis of PDAC. (a) Representative images of PDAC cancerous tissue and paired para‐cancer tissues stained with Masson trichrome (Masson); (b and c) Paired dot plots and bar graphs presented the PDAC cancerous tissue and paired para‐cancer tissues percentile values for collagen volume fraction; (d) Representative images of Masson‐stained PDAC cancerous tissue at different stages of AJCC 8th tumor stages (I, II, III, IV); (e) Bar graphs illustrated the percentile values of PDAC cancerous tissue for collagen volume fraction in different stages of AJCC 8th tumor stages (I, II, III, IV); (f) Bar graphs displayed the PDAC cancerous tissue percentile values for collagen volume fraction in different stages of the AJCC 8th TNM tumor stages; (g) Kaplan–Meier survival curve for the PDAC patients with different cancer matrix stiffness; (h) Bar graphs illustrated the PDAC patients with different stiffness of the cancer matrix.

Understanding ECM has improved rapidly with the development of technology. Collagen, glycoproteins, and proteoglycan are the main components of ECM, and their interaction determines how ECM is assembled.^20^ Collagen provides structural strength to all forms of the ECM.^21^ We performed Masson staining in human TMA containing 90 pancreatic cancer tissues and paired para‐cancer tissues with information on clinical and pathological parameters. The collagen volume fraction (CVF) was calculated as the evaluation metric of extracellular matrix thickness and tissue tension. The CVF of the cancerous tissue of PDAC was significantly higher than paired para‐cancer tissues (*p* < 0.0001) (Figure 1a–c). The CVF of cancerous tissues were positively correlated with increasing the eighth edition of American Joint Committee on Cancer staging system (AJCC 8th) N, M stage and overall stage (Figure 1d–f). Based on the CVF, we next split the patients equally into low matrix stiffness (low), mid matrix stiffness (mid), and high matrix stiffness (high) groups. We found that the high matrix stiffness group had the worst prognosis (Kaplan–Meier, log‐rank *p* = 0.0287) (Figure 1g) and the shortest median over survival (OS) (Figure 1h).

### 3.2 Matrix stiffness adjustable hydrogels characterization

**FIGURE 2:**
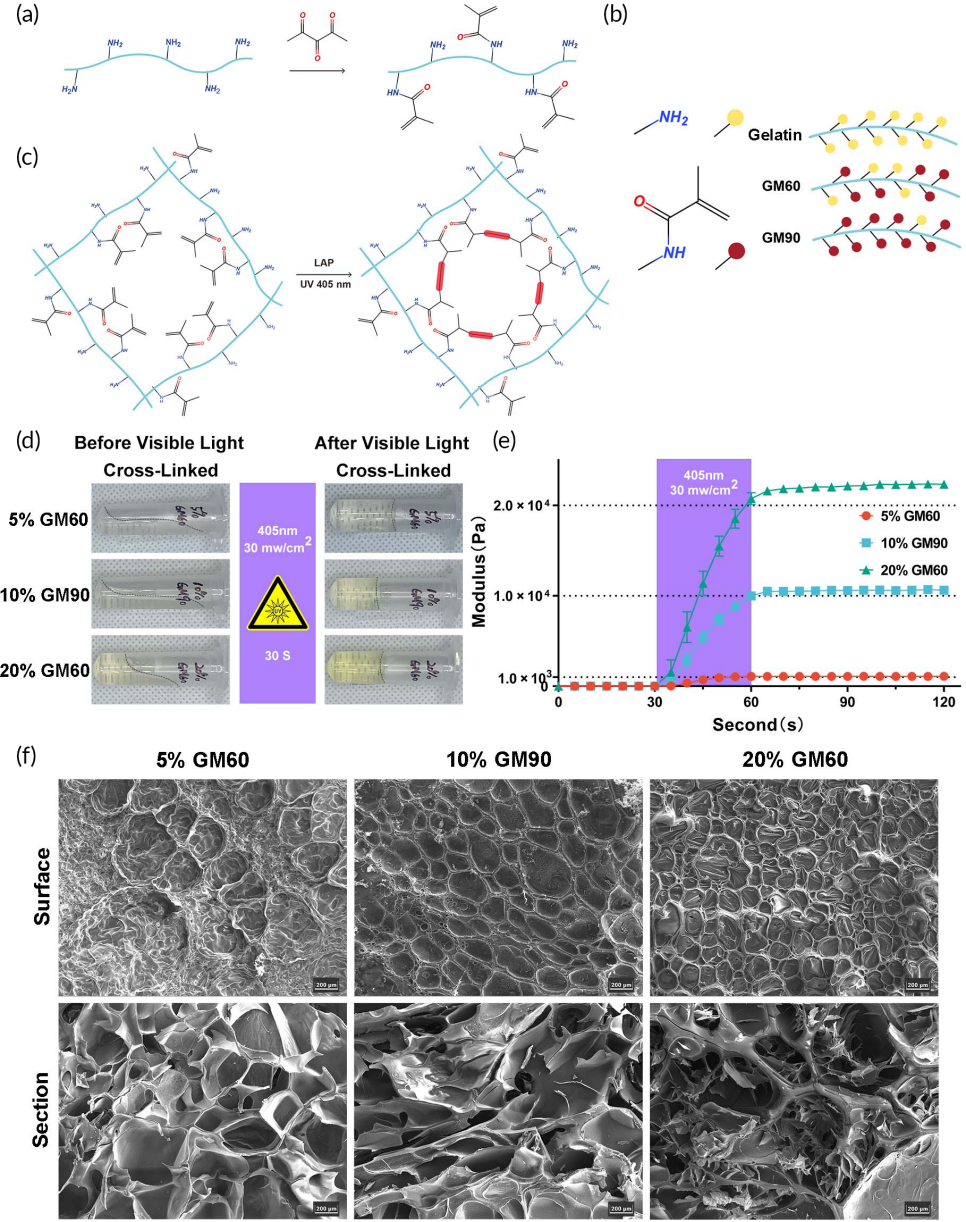
Gelatin‐methacryloyl (GelMA) hydrogel characterization. (a–c) Schematic for preparing GelMA hydrogel patch; (d) Images of hydrogels of 5% GM60, 10% GM90, and 20% GM60 from solutions after cross‐linked by visible light (405 nm) in 30 s; (e) Storage modulus (elastic modulus, *G*′, Pa) − Time (s) change curve of 5% GM60, 10% GM90, 20% GM60 hydrogels; (f) SEM of the surface and cross section of 5% GM60, 10% GM90, and 20% GM60 hydrogels (scale bar = 200 μm).

Van den Bulcke and co‐workers introduced GelMA in 2000.^22^ Due to its inherent bioactivity and physicochemical tailorability, it has gained considerable interest in tissue engineering.^14^ The reaction of methacrylic anhydride with amine and hydroxyl groups in gelatin gives rise to gelMA macromers (Figure 2a). Choosing a suitable degree of functionalization (DoF) of gelatin is the first step in the GelMA hydrogel design. By varying the methacrylic anhydride to gelatin ratio, the DoF of the synthesized GelMA batch can be customized (Figure 2b).^23^ Hydrogels can be fabricated using these macromers in the presence of light initiators, such as LAP. Through radical polymerization, gelatin chains are connected through short polymethacryloyl chains (Figure 2c). Different concentrations and DS of GelMA can be cured to form hydrogels from solutions after cross‐linking by visible light (405 nm) in 30 s (Figure 2d). The mechanical properties of the hydrogel can be readily adjusted by changing the concentrations and DS of GelMA to meet the needs of different matrix stiffness. To characterize the mechanical properties of GelMA hydrogels with different concentrations and GelMA DS, a rheological properties test was performed on samples of 5% GM60, 10% GM90, and 20% GM60, respectively. The storage modulus (elastic modulus, *G*′, Pa) – Time curve (s) presented that stiffness could be adjusted by changing the concentrations and DS (1 kPa for 5% GM60, 10 kPa for 10% GM90 and 20 kPa for 20% GM60) (Figure 2e). The fluidity before curing ensures that the biomaterial can be mixed with the cell suspension and operated easily. Stable mechanical strength after curing ensures that cells can be subjected to different mechanical forces to simulate the complex mechanical environment in vivo. Cell viability and attachment are critically influenced by the average pore size of a biological scaffold.^24^ A scanning electron microscope (SEM) was used to observe the 3D bulk structure of hydrogels. The hydrogels are highly porous with moderate thickening of the hole walls (Figure 2f).

### 3.3 In vitro matrix stiffness adjustable 3D culture system

**FIGURE 3:**
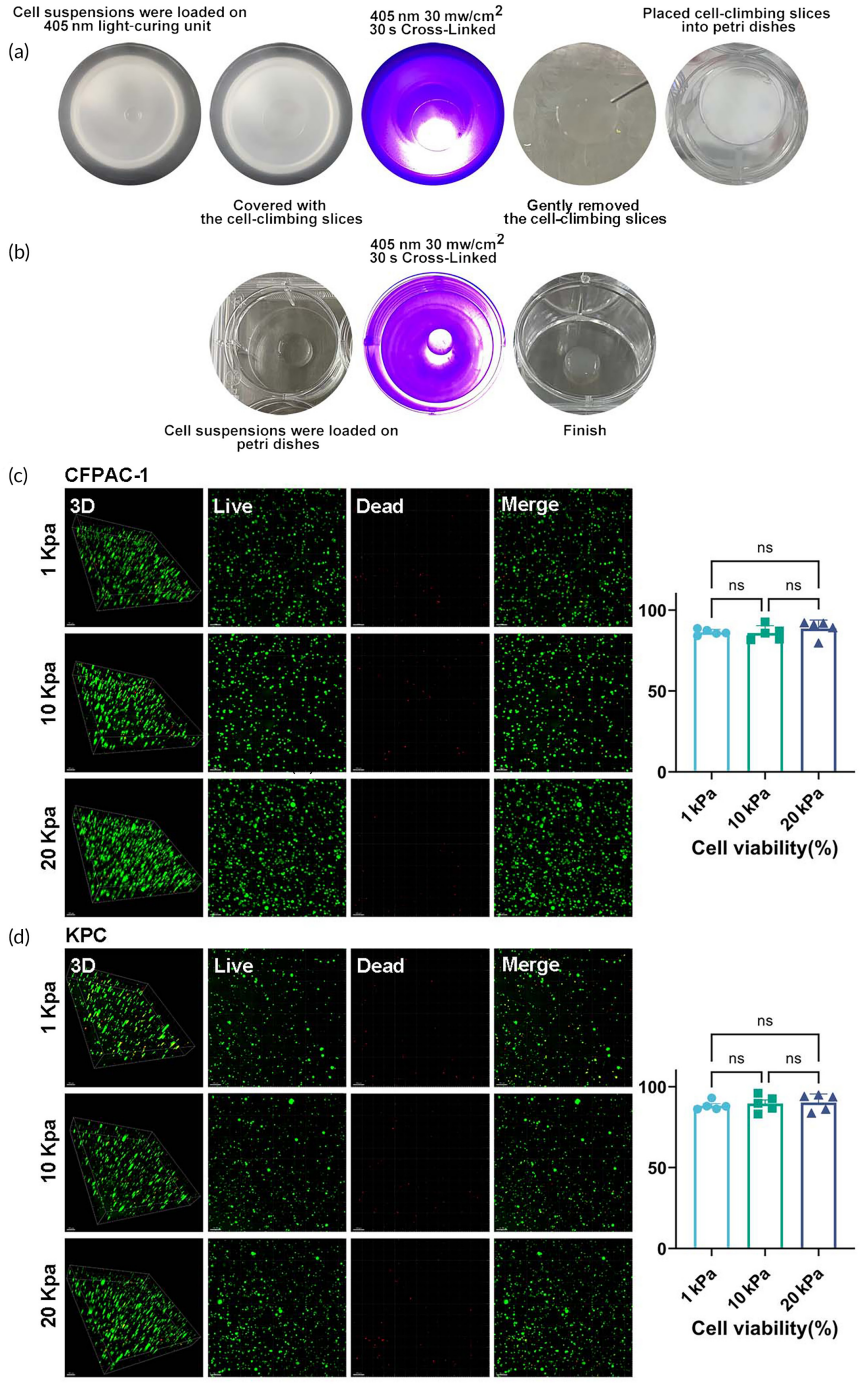
In vitro matrix stiffness adjustable 3D culture system. (a and b) Schematic representation of two methods our established for preparing in vitro 3D culture system; (c and d) Live/dead staining (Calcein/ PI staining) of CFPAC‐1 and KPC cells after culture in a GelMA 3D culture system of 1, 10, and 20 kPa for 5 days (Calcein staining as green and PI staining as red, Scale bar = 100 μm). Data presented in the graphs represent means ± SD. ***p < 0.001; **p < 0.01; *p < 0.05; ns *p* > 0.05.

We applied two methods for matrix stiffness adjustable 3D cell culture in vitro (Protocol A and Protocol B; Figure 3a,b). Protocol A can control the 3D culture thickness and its contact area with the medium by controlling the size of the cell climbing slices or the material volume. Cells can be digested faster and harvested for other experiments (effective surface area enlargement), and microscope observation is much clearer using this method. This method is suitable for experiments with certain material thickness requirements, such as shooting confocal laser scanning microscopy. The advantage of Protocol B is its simplicity and provides an environment that resembles as closely as possible that of in vivo. Cells can migrate to the medium direction (nutrient‐rich conditions) during culture. Therefore, if the experimental design is reasonable, this method can also be used to study cell migration ability.

Biocompatibility is an essential factor for the biomedical application of nanomaterials. GelMA is a promising material due to its superior biocompatibility and biodegradability.^25^ Live/dead staining confirmed that the GelMA‐based 3D culture system has excellent biocompatibility, and the cellular activity of PDAC cell lines was not significantly affected by different gelMA hydrogels of matrix stiffness (Figure 3c,d).

### 3.4 The influence of matrix stiffness on cell morphology, proliferation, and migration of PDAC cells in vitro

**FIGURE 4:**
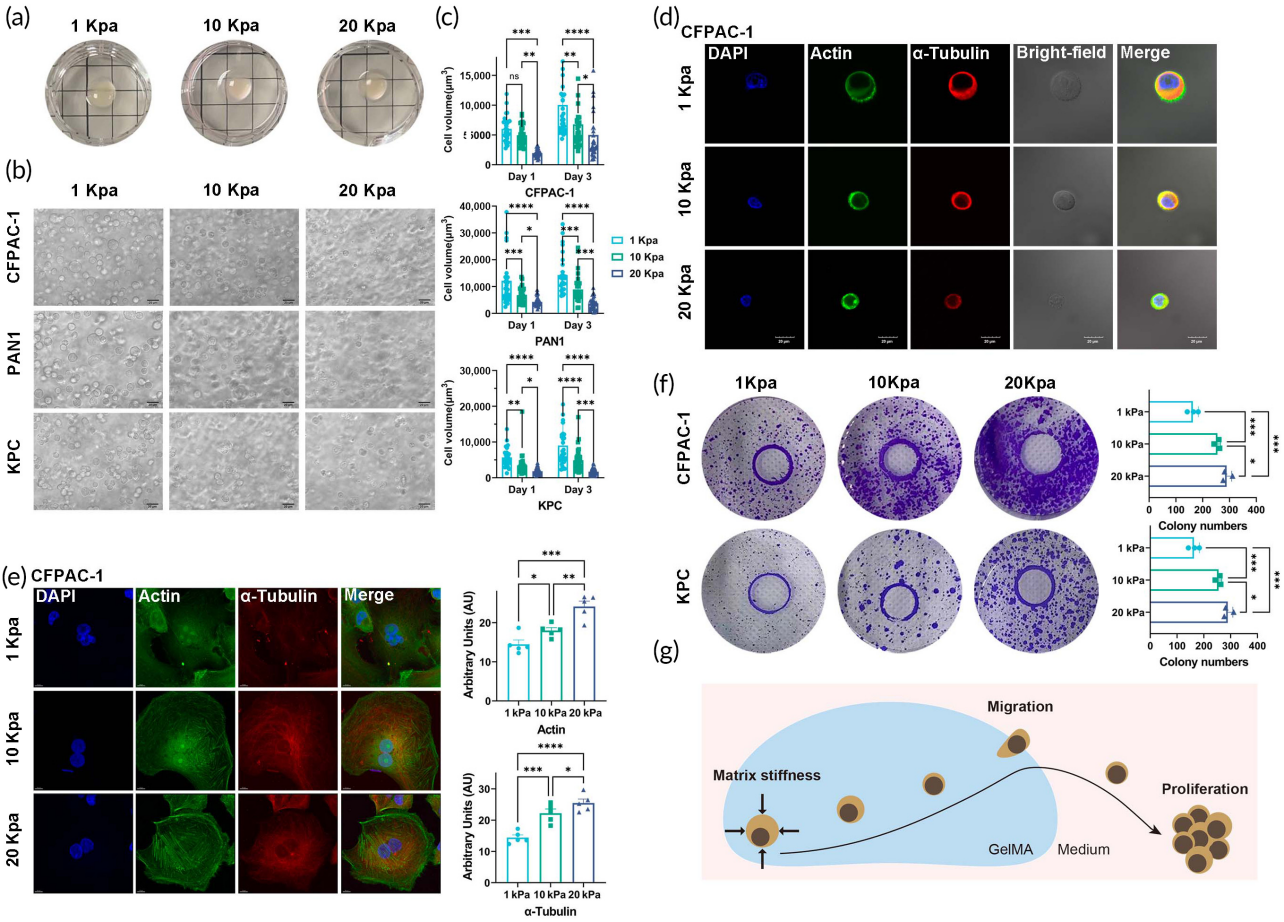
The influence of matrix stiffness on cell morphology, proliferation, and migration of PDAC cells in vitro. (a) PDAC cells encapsulated in 3D hydrogels with different matrix stiffness (1, 10, and 20 kPa); (b) Cells cultured for 5 days under 3D conditions with different matrix stiffness, cell size varies with matrix stiffness and incubation time, showing a significant gradient of change; (d and e) Increasing matrix stiffness also resulted in thickening of cytoskeletal organization stress fibrils and increased actin and α‐tubulin expression (actin staining as green and α‐tubulin staining as red); (f) The clone‐forming assay shows that changes in matrix stiffness affect cell clone‐forming abilities; (g) Schematic of a 3D cell culture system based on cross‐linked GelMA hydrogels with visible light (405 nm) cross‐linked GelMA hydrogels. Data presented in the graphs represent means ± SD. ***p < 0.001; **p < 0.01; *p < 0.05; ns *p* > 0.05.

Cellular deformation depends on the outer force minus the inner tension.^26^ The strength of external force on the cell and the strength of internal skeleton support force determine the cell shape (cell volume) under different matrix stiffness. Optical microscopic observation was employed to evaluate the cell morphology of PDAC cells at different matrix stiffnesses. Under the 3D culture conditions (Protocol B; Figure 4a), the cells showed a nearly spherical shape and cell volume decreased significantly with increased matrix stiffness (1, 10, and 20 kPa) (Figure 4b). Additionally, interestingly, after cell culture for five days, cell volume increased slightly compared to the initial state (Figure 4c). This phenomenon was most likely a combined result of the enhanced adaptability of cells to external forces (force scrambling mechanism) and swelling of the hydrogels. In addition to cell morphology, the inner tension of the cell microenvironment was recorded by staining with the cell cytoskeleton (actin and α‐tubulin). Immunofluorescent analysis of the cytoskeletal organization (actin and α‐tubulin) showed that increasing the stiffness of the matrix also resulted in thickening of the stress fibrils of the cytoskeletal organization stress fibrils and an increase in actin and α‐tubulin expression (Protocol A) (Figure 4d,e).

As described in the previous paragraphs, cells can migrate to the medium direction (nutrient‐rich conditions) during culture (Figure 4e). Through experiments similar to the principle of plate cloning, we found that the speed of cell migration is more prominent with increasing matrix stiffness. Cells migrated to culture medium are more likely to adhere and proliferate rapidly in monoclonal form (Figure 4g). Together, these results reveal that high matrix stiffness could enhance cell proliferation and migration of PDAC cells. The results also confirmed the findings of the TMA analysis (high matrix stiffness had a high ratio of regional lymph node involvement and distant metastasis) (Figure 1f).

### 3.5 The influence of matrix stiffness in vivo

Many fabrication methods have been proposed for using microgels in cell therapy, controlled drug release, and disease modeling.^15^, ^25^, ^27^, ^28^, ^29^ Because of its excellent biocompatibility (as shown above), we believe that GelMA should also be suitable as a material for in vivo experiments. Traditional orthotopic xenograft models were established by mixing cell suspension with Matrigel (BD Biosciences), and the suspension was injected into the pancreas.^30^, ^31^ Matrigel matrix is a soluble basement membrane extract isolated from Engelbreth–Holm–Swarm (EHS) mouse sarcoma rich in ECM proteins such as laminin (main component), collagen type IV, glycans heparan sulfate protein, nidogen, and various growth factors. Matrigel is expensive, the concentration and composition of each batch are not fixed, and there are strict storage and use temperature conditions. Critical Matrigel will rapidly begin solidifying near room temperature, so working quickly and keeping the Matrigel cold throughout the aliquoting process is vital. A stable and reproducible matrix stiffness gradient cannot be constructed in vivo during the experiment by Matrigel.

**FIGURE 5:**
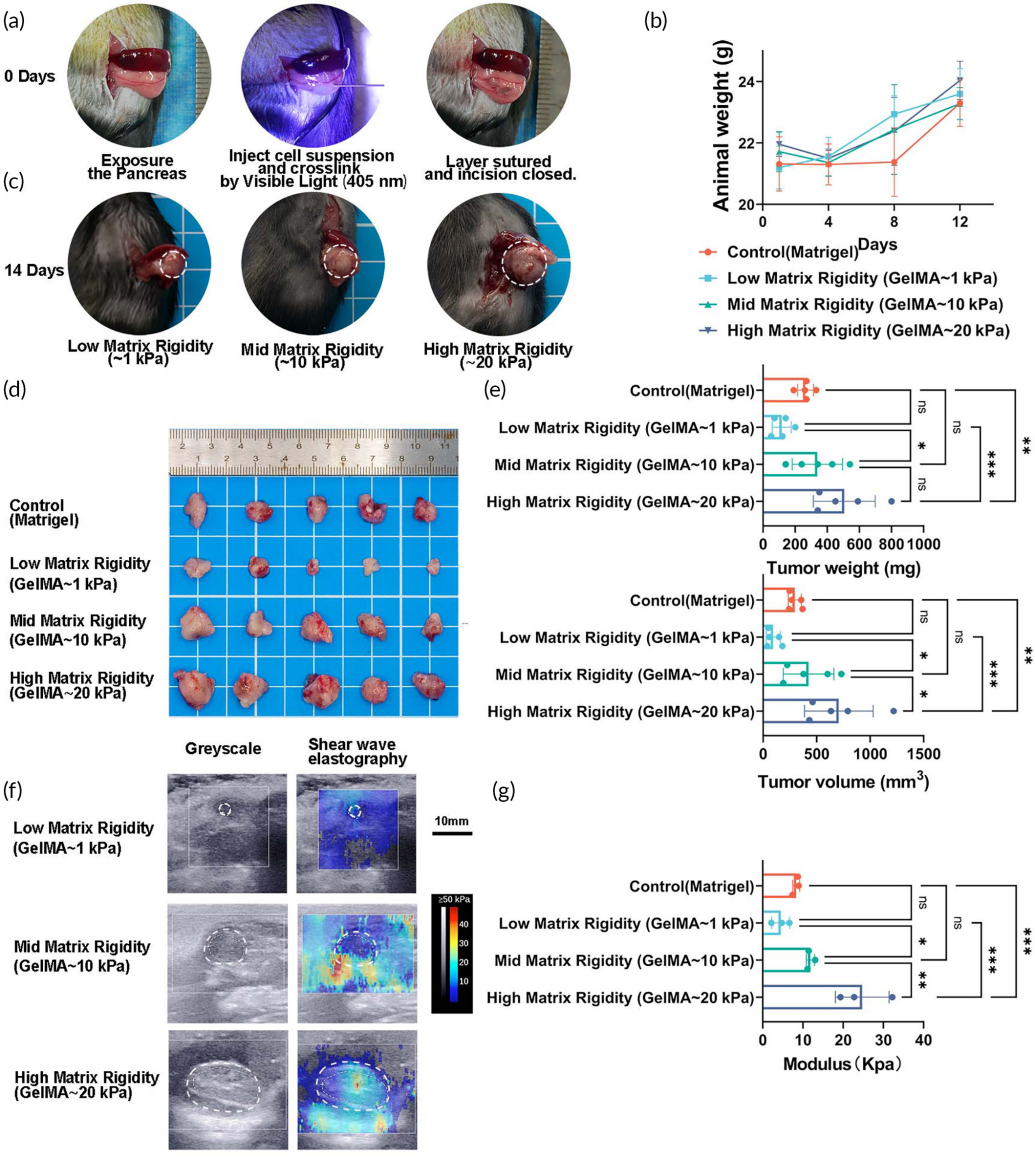
Adjustable extracellular matrix rigidity orthotopic tumor transplantation murine models of PDAC the influence of matrix stiffness in vivo. (a) Schematic representation of our established methods for preparing GelMA‐based adjustable extracellular matrix rigidity orthotopic tumor transplantation murine models; (b) Change in body weight of the mice in different groups; (c and d) Representative pictures of orthotopic tumors excised from mice; (e) Bar graph showing the average tumor volume and weight, respectively, of mice with different matrix stiffness PDAC tumors; (f) Representative pictures of greyscale and shear wave elastography ultrasound images of GelMA‐based adjustable extracellular matrix rigidity orthotopic tumor determined by noninvasive ultrasound evaluation. Side‐by‐side display of anatomical 8‐mode US image (gray image, top) and an elastography image restricted to the set study box (colored image, button) obtained with echographic 8‐mode and 2D‐SWE mode, coupled to doppler mode. The red color represents stiff tissue and the blue color reflects soft tissue (scale 0–50 kPa); (g) Bar graph showing the average tumor elastic modulus, respectively, of the orthotopic tumor of mice with different matrix stiffness PDAC tumors. Data presented in the graphs represented means ± SD. ***p < 0.001; **p < 0.01; *p < 0.05; ns *p* > 0.05.

Therefore, we tried to build an adjustable extracellular matrix rigidity model of PDAC hydrogels based on gelMA hydrogel. Figure 5a presents the general workflow for this modeling. The weight of animals weight was monitored, and no toxicity effect was observed (Figure 5b). In situ tumors have a regular tumor morphology, and matrix stiffness significantly promotes pancreatic tumor growth (Figure 5c–e). By physical palpation or using imaging modalities such as magnetic resonance imaging, computerized tomography and elastography, cancer can be detected by taking advantage of the stiffness of solid tumors compared to healthy tissue.^32^, ^33^ To study the actual tumor matrix stiffness in vivo in animal models, we measure tumor elastography in live mice using the Aixplorer SWE imaging system (SuperSonic Imagine, Aux‐en‐Provence, France) 2 weeks after modeling (Figure 5f). The results confirm that the model can effectively establish and maintain a stable and gradient matrix stiffness environment in vivo (Figure 5g). Based on tumor size, we estimated that the matrix stiffness after 1:1 dilution of Matrigel should be between the low and medium stiffness groups (1–10 Kpa), which was also confirmed by the ultrasound elastography results. Compared to Matrigel‐based models, GelMA hydrogel‐based adjustable extracellular matrix rigidity orthotopic tumor transplantation murine models have the following advantages. First, it is relatively cheap, has controlled ingredients (no growth factors), is easy to store, and can be used for short‐term storage at room temperature (37°C). Second, compared to temperature curing, light curing conditions are stable and adjustable, the batch effect is smaller, and the repeatability is higher. Third, it has no significant cytotoxicity, biological toxicity has a high tumor formation rate and can build and maintain a stable matrix stiffness microenvironment.

### 3.6 Multiple signaling pathway pathways are involved in the regulation of stiffness‐dependent PDAC progression

**FIGURE 6:**
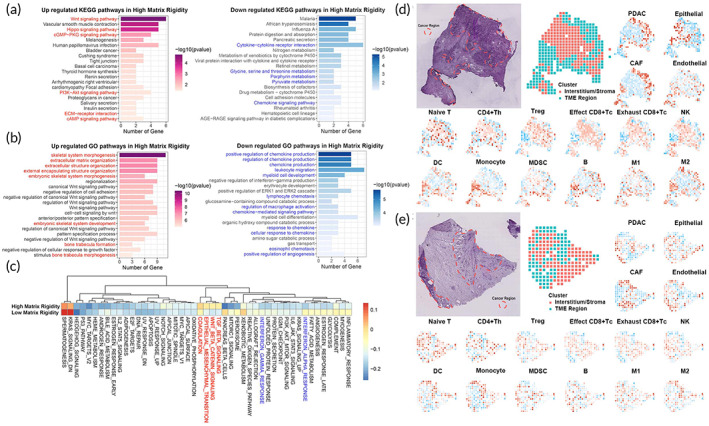
Bulk RNA‐seq and space transcriptome to the mechanisms of stiffness‐dependent PDAC progression. Adjustable orthotopic extracellular matrix rigidity tumor transplantation murine models of PDAC were built in immune‐competent C57BL/6J mice. After 2 weeks, tumor tissues were collected for bulk RNA‐seq assay. (a) Bar plot showing KEGG enrichment analysis of the DEPs (upregulated and downregulated); (b) Bar plot showing GO enrichment analysis of the DEPs (upregulated and downregulated); (c) The heat maps representing the GSVA analysis based on Hallmark gene sets; (d and e) Space transcriptome mapping immune infiltration in the pancreatic ductal adenocarcinoma microenvironment using public data. Including original tissue images stained with HE (marked by the pathologist and showing the cancer foci and stroma), the cancer foci and stroma shown by spatial transcriptome analysis, and the spatial distribution of the various cell types (red shows enrichment) mapped based on spatial transcriptome data analysis.

To explore the mechanisms of stiffness‐dependent PDAC progression, we performed a bulk RNA sequencing using tumor tissues of orthotopic tumor transplantation murine (low matrix rigidity vs. high matrix rigidity). Orthotopic tumors with high matrix rigidity of PDAC had a distinguished transcriptomic profile. KEGG and GO enrichment analyses were performed based on the identified DEGs and GSVA analyses based on Hallmark gene sets. Enrichment analyses showed that high matrix rigidity tumor microenvironment (TME) was highly associated with extracellular matrix organization, multiple signaling pathway pathways, and immune response (Figures 6a–c). The results illustrated that Wnt signaling pathway, Hippo signaling pathway, PI3K− Akt signaling pathway, epithelial‐mesenchymal transition (EMT), TGFβ signaling. cancer‐related pathways were upregulated in the high matrix rigidity group. Extracellular matrix organization, skeletal system morphogenesis. stiffness‐related pathways were also upregulated. An immune response such as leukocyte migration, response to chemokine, chemokine production, cytokine−cytokine receptor interaction, interferon alpha response, and interferon‐gamma response was downregulated in the high matrix rigidity group.

To further corroborate the role of matrix rigidity in tumor immunosuppressive microenvironment, we analyzed immune infiltration in pancreatic ductal adenocarcinoma microenvironment using public ST data.^19^ Consistent with the bulk RNA‐seq results above, in terms of spatial location, the extracellular matrix significantly inhibits the infiltration of anti‐tumor immune cells (effect T, NK, M1) into the tumor area, while the distribution of cells that promote immunosuppression (M2, CAF, monocyte) is not affected by the matrix, even significantly colocalized with tumor cells (Figure 6d,e).

Both transcriptomic and public ST data indicated that stiffness was closely related to PDAC progression and anti‐tumor immunosuppression.

### 3.7 Extracellular matrix rigidity promotes EMT and the tumor immunosuppression microenvironment

**FIGURE 7:**
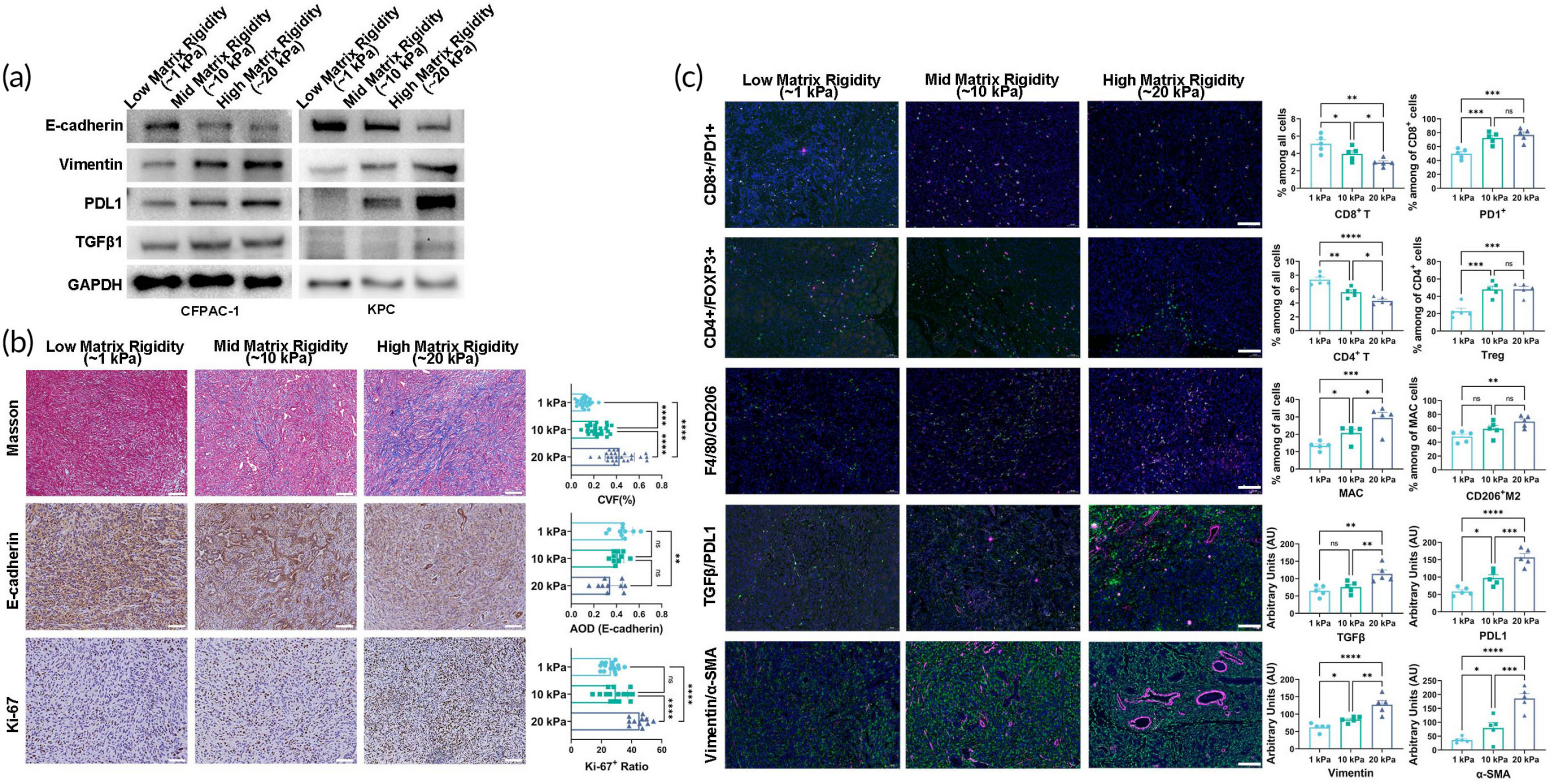
Extracellular matrix rigidity promotes EMT and the tumor immunosuppression microenvironment. To further confirm the findings from the bioinformatics analysis, we validated protein levels and difference in immune cell infiltration in vitro and in vivo. (a) Immunoblotting analysis of EMT (E‐cadherin, Vimentin), TGFβ1, and PDL1 expression in PDAC cells cultured in different matrix rigidity 3D culture systems (1–20 Kpa); (b) Multiplex IHC staining to assess collagen production level (Masson staining), EMT status (E‐cadherin) and cell proliferation (Ki‐67) in different matrix rigidity murine PDAC tumor tissues; (c) Multiplex IF staining to map the TME of in different matrix rigidity murine PDAC tumors tissues include CD8^+^ T cells (CD8^+^), Exhaust CD8^+^ cytotoxic T (CD8^+^PD1^+^), CD4^+^ T cells (CD4^+^), regulatory T cells (Treg), macrophages (F4/80^+^), M2 macrophages (F4/80^+^CD206^+^), EMT status (Vimentin and α‐SMA), and TGFβ1/PDL1 expression.

Increasing evidence points to the role of EMT in fibrosis, cancer progression, metastasis, and drug resistance.^34^, ^35^ TGF‐β was a pleiotropic cytokine and modulated various physiological processes, including immunological reaction, cell proliferation, and EMT. TGF‐β promoted tumor growth by inhibiting antitumor immunoreaction in the tumor microenvironment and promoted tumor metastasis via EMT.^36^ We further assessed EMT status, TGFβ1, and PDL1 expression in the 3D culture system model in vitro by western blot (Figure 7a). EMT, TGFβ1, and PDL1 expression increased with the increase of matrix stiffness. In vivo, we perform Masson staining, IHC, and IF to assess the role of matrix rigidity in fibrosis, cancer progression, EMT, and immunosuppression (Figure 7b,c). Masson staining showed that extracellular matrix rigidity effectively improved extracellular matrix organization. Anti‐E‐cadherin, anti‐vitimentin, and anti‐α‐SMA staining showed that the rigidity of the extracellular matrix effectively enhanced EMT. Anti‐CD8, anti‐PD1, anti‐CD4, anti‐FOXP3, anti‐F4/80, anti‐CD206, anti‐TGFβ1 and anti‐PDL1 IF staining showed that extracellular matrix rigidity effectively improved immunosuppression through decreased tumor‐infiltrating anti‐tumor lymphocyte (CD8^+^ T, CD4^+^ T). Tumor immunosuppression‐related cells (Treg, CD206^+^ M2) and marker protein (TGFβ1 and PDL1) were also enhanced in the high matrix rigidity group.

## 4 CONCLUSIONS

Matrix stiffness is strongly associated with a poor prognosis in PDAC. In this study, by adjusting GelMA concentration and DS, a novel adjustable extracellular matrix rigidity tumor model based on visible light (405 nm) cross‐linked hydrogels was designed for in vitro and in vivo PDAC experiments. The GelMA‐based hydrogel has porous, adjustable mechanical properties and excellent in vitro and in vivo biocompatibility. The GelMA‐based in vitro 3D culture method can effectively form a gradient and stable extracellular matrix stiffness, affecting cell morphology, cytoskeleton remodeling, and malignant biological behaviors such as proliferation and metastasis. Furthermore, in vivo evaluation confirmed that this model is suitable for in vivo studies with no significant toxicity. High matrix stiffness can significantly promote PDAC progression and immunosuppression.

## AUTHOR CONTRIBUTIONS

**Haoxiang Zhang:** Conceptualization (lead); data curation (lead); Study design (lead); methodology (lead); collection, analysis, interpretation of data (lead); visualization (lead); writing–original draft (lead). **Jiaoshun Chen:** Study design (equal); methodology (equal); collection, analysis, interpretation of data (equal); visualization (equal); writing–original draft (equal). **Xiaoqing Hu:** Methodology (equal); collection, analysis, interpretation of data (equal), interpretation of data (equal); visualization (equal); writing–original draft (equal). **Jianwei Bai:** Collection, analysis, interpretation of data (equal); resources (equal); validation (equal); formal analysis (equal). **Tao Yin:** Supervision (lead); funding acquisition (lead); conceptualization (lead); methodology (equal); validation (equal); writing–review & editing; writing–original draft (lead).

## FUNDING INFORMATION

This work was supported by the National Natural Science Foundation of China (81772564 and 82173196) and Key Research and Development Program of Hubei (2022BCA012).

## CONFLICT OF INTEREST STATEMENT

The authors declare no conflicts of interest.

### PEER REVIEW

The peer review history for this article is available at https://www.webofscience.com/api/gateway/wos/peer-review/10.1002/btm2.10518.

## Supporting information


**Table S1.** Baseline characteristics of the tissue microarray.
**Table S2.** The lists of cell gene markers

## Data Availability

All data used to support the findings of this study are available from the corresponding author upon request.
