# Supplementary material for: Adjustable extracellular matrix rigidity tumor model for studying stiffness dependent pancreatic ductal adenocarcinomas progression and tumor immunosuppression
Source: Bioeng Transl Med. 2023 Apr 5;8(3):e10518. doi: 10.1002/btm2.10518 (PMC10189475; doi:10.1002/btm2.10518)
Supplement: Supplementary file 1 — Table S1. Baseline characteristics of the tissue microarray. Table S2. The lists of cell gene markers [file BTM2-8-e10518-s001.docx]

**Table S1. Baseline characteristics of the tissue microarray.**

|  | Total(n=90) | Low(n=30) | Mid(n=30) | High(n=30) |
| --- | --- | --- | --- | --- |
|  | n(%) or mean±SD | n(%) or mean±SD | n(%) or mean±SD | n(%) or mean±SD |
| Age | 57.53±8.70 | 58.97±8.73 | 56.30±7.84 | 57.33±9.53 |
|  |  |  |  |  |
| Sex |  |  |  |  |
| Female | 37(41.1) | 8(26.7) | 15(50) | 14(46.7) |
| Male | 53(58.9) | 22(73.3) | 15(50) | 16(53.3) |
|  |  |  |  |  |
| AJCC8th |  |  |  |  |
| Grade |  |  |  |  |
| Ⅰ | 23(25.6) | 18(60) | 2(6.7) | 3(10) |
| Ⅱ | 28(31.1) | 6(20) | 15(50) | 7(23.3) |
| Ⅲ | 16(17.8) | 3(10) | 8(26.7) | 5(16.7) |
| Ⅳ | 23(25.6) | 3(10) | 5(16.7) | 15(50) |
| T stage |  |  |  |  |
| T1 | 7(7.8) | 5(16.7) | 1(3.3) | 1(3.33) |
| T2 | 48(53.3) | 19(63.3) | 13(43.3) | 16(53.3) |
| T3 | 23(25.6) | 4(13.3) | 10(33.3) | 9(30) |
| T4 | 11(12.2) | 2(6.7) | 5(16.7) | 4(13.3) |
| N-stage |  |  |  |  |
| N0 | 32(35.6) | 21(70) | 5(16.7) | 6(21.4) |
| N1 | 45(50) | 8(26.7) | 19(63.3) | 18(64.3) |
| N2 | 11(12.2) | 1(3.3) | 6(20) | 4(14.3) |
| M stage |  |  |  |  |
| M0 | 66(73.3) | 27(90) | 24(80) | 15(50) |
| M1 | 24(26.7) | 3(10) | 6(20) | 15(50) |
|  |  |  |  |  |
| Survival | Mean±SE | Mean±SE | Mean±SE | Mean±SE |
|  | 17.59±0.99 | 20.73±1.72 | 17.36±1.69 | 14.49±1.47 |
|  | Median±SE | Median±SE | Median±SE | Median±SE |
|  | 16.5±0.95 | 18.00±2.40 | 15.10±2.74 | 12.00±3.01 |

**Table S2. The lists of cell gene markers**

| Cell Type | Gene list |
| --- | --- |
| CD4T | RPS27,RPS27A,RPSA,RPL34,IL7R,LTB,RPS18,RPS10,RPS12,GIMAP7,RPS6,MS4A1,PTPRCAP,CD3D,LDHB,RPS29,RPS15A,CCR7,RPS21 |
| CD8_EF | GNLY,CD8A,DUSP2,GZMK,GZMA,PRF1,CTSW,PTPRCAP,CD3E,CD3D,CD3G,GZMH,GZMB,CCL5,CCL4,CST7,GZMM,HCST,NKG7 |
| CD8_EX | AC092580.4,ZFP36L2,CD8A,CD8B,CXCR4,CYTIP,IL7R,PRDM1,SYTL3,CD3E,CD3D,CD3G,CD69,KLRC1,BTG1,GZMH,CCL5,CCL4,CD7 |
| CD8T | AC092580.4,GNLY,CD8A,CD8B,DUSP2,CXCR4,IL7R,GZMK,GZMA,PTPRCAP,CD3E,CD3D,CD3G,CD69,GZMH,CCL5,CCL4,HCST,NKG7 |
| iFIB | FN1,IGFBP5,COL6A3,APOD,IGFBP7,CXCL14,SPARC,COL1A2,CTHRC1,PTGDS,MGP,LUM,DCN,SERPINF1,COL1A1,MMP11,FBLN1,SFRP2,SFRP4 |
| M1 | S100A9,S100A12,S100A4,VCAN,LST1,AIF1,FCN1,LYZ,FGL2,MS4A6A,MPEG1,COTL1,CD36,CYBB,CFP,LGALS2,DUSP6,CPVL,AP1S2 |
| M2 | MARCO,SLC11A1,CXCL3,SPP1,VCAN,GPNMB,CTSB,FABP5,PLIN2,CTSL,CTSD,RNASE1,NUPR1,CD68,CCL2,APOE,APOC1,FTL,CSTB |
| MDSC | S100A9,S100A8,MNDA,FCGR3B,RGS2,G0S2,GCA,CXCR2,BASP1,DUSP1,SOD2,NAMPT,SLC25A37,SRGN,IFITM2,SORL1,FOS,CMTM2,FPR1 |
| Mo | C1QC,C1QB,CD14,CD74,AIF1,HLA-DRA,HLA-DRB5,HLA-DRB1,HLA-DQA1,HLA-DQB1,HLA-DQA2,HLA-DMA,HLA-DPA1,HLA-DPB1,MS4A6A,MS4A7,LYZ,MAFB,APOE |
| myFIB | SOD3,IGFBP7,SPARCL1,CALD1,TPM2,ADIRF,ACTA2,C11orf96,TAGLN,MCAM,NDUFA4L2,CSRP2,MYH11,MT2A,MT1E,MT1M,DSTN,MYL9,PPP1R14A |
| NEU | FCGR3B,PTGS2,RGS2,MXD1,IL1R2,NABP1,ABTB1,LUCAT1,NCF1,NAMPT,SLC25A37,MTRNR2L8,NEAT1,SORL1,LRRK2,SMCHD1,MYO1F,CYP4F3,MMP9 |
| nFIB | FN1,IGFBP5,COL6A3,IGFBP7,SPARC,COL1A2,CALD1,CTHRC1,LUM,DCN,POSTN,COL1A1,COL6A2,SFRP2,COL6A1,MMP11,MGP,PTGDS,CXCL14 |
| PDAC | MUC1,EPCAM,CLDN18,TM4SF1,S100P,HSP90AB1,CLDN4,MAL2,LCN2,KRT7,IFI27,KRT19,WFDC2,FXYD3,C19orf33,CEACAM5,CEACAM6,TFF2,TFF1 |
| T_NAIVE | RPS27A,RPL32,RPSA,RPL34,RPS3A,IL7R,TCF7,LTB,RPS18,RPS12,GIMAP7,RPS6,PRKCQ-AS1,CD3D,LDHB,RPS29,RPS15A,CCR7,NOSIP |
| Treg | TNFRSF4,CD2,CTLA4,TIGIT,LTB,FOXP3,IL2RA,RTKN2,SPOCK2,BIRC3,CD3D,ETS1,CD27,TBC1D4,BATF,IL32,LAIR2,DUSP4,UGP2 |
| DC | C1QB,SPP1,HLA-DRB5,GPNMB,CTSD,APOE,APOC1,FTL,CD68,CHIT1,ACP5,FABP5,CTSZ,LIPA,NUPR1,HBA1,FBP1,HBA2,HBB |
| Endo | EPAS1,SDPR,HYAL2,SPARCL1,SPRY1,GNG11,STC1,EGFL7,VWF,A2M,FLT1,SLC9A3R2,CD320,F2RL3,TIMP3,RAMP2,PLVAP,CLDN5,FABP5 |
| NK | C1orf186,GNLY,IL1RL1,GATA2,CPA3,KIT,AREG,HPGDS,GZMA,LTC4S,CLU,PRF1,SLC18A2,CTSW,VWA5A,GZMB,TPSAB1,NKG7,CTSG |
| CAF | FN1,IGFBP5,APOD,IGFBP7,SPARCL1,SPARC,COL1A2,CALD1,TPM2,ADIRF,ACTA2,C11orf96,TAGLN,MGP,LUM,DCN,COL1A1,MYL9,SFRP2 |
| EPI | CELA2A,CELA2B,CELA3B,CELA3A,AMY2B,AMY2A,REG1A,CPB1,CLPS,CPA2,CPA1,PRSS1,CEL,PNLIP,PLA2G1B,GP2,CTRB2,CTRB1,SYCN |
| B | ITM2C,IGJ,SPCS3,MZB1,PRDX4,SSR4,FKBP2,SPCS2,FKBP11,HSP90B1,RP11-731F5.2,TNFRSF17,HERPUD1,SEC11C,CD79A,SDF2L1,IGLL5,DERL3,XBP1 |
